# Real-world data on canine chronic kidney disease in Greece: clinical and quality of life insights

**DOI:** 10.3389/fvets.2025.1601044

**Published:** 2025-08-11

**Authors:** Ioulia Chortara, Constantina N. Tsokana, Eleni Pavlidou, Thaisa L. Sandri, Andrea Wright, George Valiakos

**Affiliations:** ^1^Asclepius One Health Platform, Athens, Greece; ^2^Faculty of Health Sciences, School of Veterinary Medicine, Aristotle University of Thessaloniki, Thessaloniki, Greece; ^3^Global Medical Affairs, Zoetis Inc., Parsippany, NJ, United States; ^4^Faculty of Veterinary Medicine, School of Health Sciences, University of Thessaly, Karditsa, Greece

**Keywords:** animal health, canine, chronic kidney disease, disease management, Greece, observational study, quality of life, real-world data

## Abstract

**Introduction:**

Chronic Kidney Disease (CKD) is an important health concern in dogs, characterized by structural and functional kidney abnormalities persisting for over three months. Despite being significant for dog health and commonly managed by veterinarians, there is a lack of real-world data (RWD) on canine CKD (cCKD) worldwide. This study aimed to address this gap by collecting and analyzing RWD on cCKD cases from Greece.

**Methods:**

An observational study was conducted across Greece from January to October 2023. Data were collected from 215 dogs diagnosed with cCKD, staged according to the International Renal Interest Society (IRIS) criteria. Veterinarians and owners completed detailed forms capturing clinical, demographic, and Quality of Life (QoL) information. Laboratory analyses included serum biochemistry and urinalysis. Canine QoL was assessed using the VetMetrica™ health-related quality of life (HRQL) instrument. Statistical analyses were performed to identify trends and correlations.

**Results:**

Most cases (79.6%) were classified as early IRIS stages (1 and 2) and 20.5% in advanced stages (3 and 4). Smaller sized dogs and mixed breeds represent approximately 50 and 38% of the enrolled cases, respectively. Mean age of cCKD diagnosis was 10.7 years. Statistical analysis showed that larger-sized dogs develop CKD at younger ages and approximately one quarter of reported cases fall below the geriatric age. Common comorbidities included cardiovascular (28.8%), periodontal (26.5%), degenerative joint disease (18.1%), and liver disease (17.2%) which had a significant impact on the QoL of the animal even in early IRIS stages. Clinical signs such as polyuria, vomiting, diarrhea and halitosis were more prevalent in cases with advanced IRIS stages and were reported as troublesome by owners.

**Conclusion:**

This study provides valuable RWD on cCKD in Greece, highlighting the importance of early detection and comprehensive management, as it can lead to more effective treatment plans, reduce the disease progression, and ultimately improve the overall well-being of the dogs. Moreover, data analysis demonstrates the significance of comorbidities and their impact on the QoL of a dog with cCKD; addressing comorbidities early, longevity and quality of life for canine companions can be enhanced. Future research should expand on these insights to enhance global understanding and management of cCKD.

## 1 Introduction

Chronic Kidney Disease (CKD) represents a significant health concern in dogs, with progression rates that vary widely among cases. This condition is characterized by structural or functional abnormalities in one or both kidneys, persisting for an extended period, typically three months or more (1). The gradual decline in renal function often leads to severe health implications and reduced survival rates. To this end, the mortality rate increases in dogs in advanced CKD stages (2).

Canine CKD (cCKD) is a multifactorial condition influenced by genetic, environmental, and pathological factors. The etiology of cCKD encompasses a range of underlying causes, including genetic predispositions, age-related changes, and systemic diseases (3). Several risk factors contribute to its development and progression including advanced age, history of anesthetic-surgical procedures, cardiac and endocrine disorders, tumors, exposure to nephrotoxic drugs, and infectious diseases (4).

The prevalence of cCKD in the general population can vary between 0.5 to 3.0% and can reach 10.0% in the hospitalized dogs (2, 5); however, it has been suggested that in certain high-risk breeds, prevalence rates can reach as high as 25% (1, 6). Moreover, certain breeds, such as Cocker Spaniels, exhibit higher susceptibility to cCKD, while genetic predispositions have been identified in breeds like Boxers (1, 3).

Besides the health considerations mentioned above, cCKD poses additional diagnostic and management challenges, necessitating a comprehensive approach encompassing early detection, dietary management, and innovative therapeutic strategies (4). Systematic screening for at-risk animals can facilitate early disease diagnosis and improve treatment outcomes. In this context, the International Renal Interest Society (IRIS) has developed a four-stage scoring system for canine and feline CKD. This system utilizes fasting blood creatinine and symmetric dimethylarginine (SDMA) concentrations, urine protein to creatinine ratio (UP/C), and blood pressure measurements to classify cCKD cases (www.iris-kidney.com), supporting diagnosis, treatment, prognosis, and research efforts (7).

Another aspect of cCKD that is not much researched and discussed in the literature concerns its impact, as a chronic disease, on dogs’ welfare and quality of life (QoL). As with human patients, dogs with cCKD experience a decrease in QoL in comparison to healthy individuals, worsening even more with the severity and progression of the disease. Assessing canine QoL is essential to provide a comprehensive understanding of both physical and emotional wellbeing, also enabling veterinarians and owners to make informed decisions about treatment and disease management. In addition, it is currently known that chronic diseases in pets impact the well-being of their owners, leading to various emotional, social, and practical challenges. A greater burden, stress and signs of depression/anxiety, as well as poorer QoL, in owners of companion animals with chronic or terminal disease compared to owners of healthy pets have been shown previously (8). However, studies also indicate that pets enhance mental well-being (9) and offer social support (10), and Ηuman-Αnimal Ιnteractions (HAI) can alleviate signs of anxiety and depression in pet owners (11). Notably, a study conducted in the USA showed that ownership provided dog owners with emotional support and companionship irrespective of the cCKD stage of their pet (12). With this information, it becomes apparent that from a One Health perspective, the mental health of pet owners and the health of pets are interconnected (13) and deserve to be further explored.

In the era of evidence-based medicine, real-world data (RWD) has gained ground in human medicine (14) and is now becoming relevant in veterinary research (12) due to its ability to capture diverse health outcomes and responses to treatment. These data are collected outside randomized controlled trials, in a real-world context, offering a comprehensive view of patient health and healthcare delivery. Data comes from multiple sources such as medical records, insurance claims, and research-specific datasets (15).

Integrating RWD in veterinary research can enhance clinical decision-making, improve the accuracy of health assessments and provide comprehensive insights into disease management, including chronic diseases such as cCKD. There is a lack of RWD on cCKD in many countries worldwide despite this disease’s importance and devastating nature if left unmanaged for dogs.

Using a RWD collection system for dogs developed by the Asclepius One Health platform and implemented in Greece, this study aims to provide a comprehensive analysis of the clinical characteristics and comorbidities of dogs with cCKD. By assessing the impact of these factors on dogs’ QoL, the study seeks to offer valuable insights that could enhance veterinary care and improve the well-being of canine patients.

## 2 Materials and methods

### 2.1 Development of data collection forms

As a first step, forms were developed to systematically collect data for cases of cCKD. The data to be collected were decided by the study team through literature review of similar studies referring to kidney diseases of both humans and animals (1, 16–18). The following data forms were finally included:

#### 2.1.1 Screener case report form

Designed to confirm each case’s eligibility for study inclusion. It contained specific inclusion and exclusion criteria that the cCKD case needed to meet (Supplementary Table A1). Additionally, it documented the IRIS stage of the animal, as classified by the veterinarian, based on the dog’s medical history and clinicopathological findings, and following the IRIS guidelines (Supplementary Table A2). The study enrolled client-owned dogs, aged three years or older with a stable, veterinarian-confirmed diagnosis of cCKD at any IRIS stage. The primary caretaker of the dog had to be over 18 years old and able to complete the study questionnaires in Greek. Dogs were excluded if they were pregnant, lactating, on an unbalanced diet, or had been hospitalized within the seven days prior to enrollment. Additional exclusion criteria targeted specific comorbidities that could interfere with the assessment of cCKD, such as acute kidney injury, various endocrine disorders (e.g., uncontrolled hyperparathyroidism), specific congenital or obstructive renal conditions, and any suspected or confirmed malignancy. Dogs were also excluded if they had recently been treated for active parasitic or infectious diseases known to cause kidney damage (e.g., canine leishmaniosis, leptospirosis) or were concurrently enrolled in another clinical trial involving an investigational product.

#### 2.1.2 Veterinarian-completed form

Designed to collect essential demographic characteristics of the canine patient including the sex, age, breed, weight, and any concurrent health conditions (Supplementary Table A3).

#### 2.1.3 Veterinarian/owner completed case form

This case form was collaboratively completed by both the veterinarian and the dog’s owner. It was designed to record details on the dog’s diet, and medical history and it also included a checklist of current clinical signs of the animal as well as an assessment of which were considered the most troublesome by the owner for himself as well as his dog (Supplementary Tables A4, A5).

#### 2.1.4 Owner-completed form

Designed to anonymously collect basic demographic information from the dog owners who participated in the study (Supplementary Table A6).

#### 2.1.5 The VetMetrica health-related quality of life (HRQL) questionnaire

The canine version of the general VetMetrica™ instrument was used to measure HRQL in dogs included in the study (19, 20). VetMetrica™ is a HRQL instrument which contains 22 behavior-based items to which the dog owner responds using a 7-point Likert scale (0 = Not at all to 6 = Could not be more). To avoid potential bias, owners conducted their assessment on the same day but independent of the practitioner’s clinical examination. This resulted in scores in four HRQL domains including Energetic/enthusiastic (E/E), Happy/Content (H/C), Active/Comfortable (A/C), and Calm/Relaxed (C/R). Summary scores in physical wellbeing (PWB) and emotional wellbeing (EWB) were calculated by averaging the E/E and A/C scores (PWB) and H/C and C/R scores (EWB). To aid interpretability of the tool, raw domain scores (0–6) were normalized and displayed on a 0–100 scale (21, 22).

### 2.2 Participant recruitment

The data collection process was conducted by Greek veterinarians affiliated with the Asclepius One Health (AOH) platform; an independent non-profit organization based in Greece with international operations, that has previously performed similar data collection studies from veterinary practitioners (23).

The communications were conducted by veterinarians who were members or affiliates of AOH, comprising private practitioners and academics. The veterinarians initially invited to participate in the project were selected from the professional network of AOH members, based on their geographical distribution across Greece. Initial contact was established via telephone, followed by written correspondence through electronic mail, wherein data forms were transmitted, and the study’s purpose and methodology were elucidated in writing. Consequently, a network of veterinarians was established throughout Greece, with the objective of examining their clinical records for dogs with cCKD, staged according to the IRIS system.

Veterinarians contacted owners of eligible dogs to extend an invitation to participate in the study, aiming to include a diverse sample based on age, sex, breed, and IRIS stage. Prior to enrollment, all participants provided written informed consent.

### 2.3 Laboratory analysis

To assist with cCKD diagnosis and precise IRIS staging, practitioners were encouraged to collect serum and urine samples (at their discretion) at two Timepoints [Timepoint 1 (Day 0–7) and Timepoint 2 (Day 30–37)] concurrently with the completion of the data collection forms. Samples were obtained on a routine basis from dogs for which the veterinarian intended to request a blood biochemistry and urinalysis test.

To ensure accuracy and consistency, practitioners were asked to send collected samples to an ISO 17025-accredited and ISO 9001 certified laboratory in Athens, Greece (Vet in Progress Plus), equipped with advanced analytical instruments that qualify the accuracy of the measurement data (24, 25).

In serum samples, blood creatinine (bCREA) was measured using an enzymatic assay, which minimizes interference from bilirubin, hemoglobin, and various drugs (26, 27). The analysis also included blood urea nitrogen (BUN), albumin (ALB), total proteins (TP), phosphorus (P), calcium (Ca and Ca++), potassium (K), sodium (Na), chloride (Cl), and the Na/K ratio. In urine samples, urinalysis was conducted to measure urinary creatinine (uCREA), urinary protein (uP), and the urinary protein-to-creatinine ratio (UPCR). Urine testing was optional and, where possible, urine was obtained by cystocentesis.

### 2.4 Data collection, database development

Data forms were provided in pen and paper format to the practitioners. Various options were provided to the practitioners for submitting the completed data forms, according to their preferences: (1) Data upload in a Veterinary Management Platform (VetPlatform, https://vetplatform.gr/en_gb/) where the data forms were available, (2) scanned, via e-mail, (3) via postal delivery, and (4) direct submission during in-person visits of members of the study team. Data was extracted and transferred to specifically designed spreadsheets, which incorporated columns with macro-drop-down lists for each possible response or a free-text field for open-ended questions.

### 2.5 Weight classification, age considerations and comorbidity grouping

As average weight in adult dogs is associated with specific breed sizes, the weight variable was transformed into categorical by establishing five weight groups (WGs); Very Small (<6.75 kgs), Small (6.75–<11.25 kgs), Medium (11.25–<20.25 kgs), Standard (20.25–29.25 kgs) and Large >29.25 kgs, similarly to other studies approach (28).

Building on the classification by weight, geriatric age was also considered in relation to breed size, as it varies significantly across categories. According to the UC Davis Book of Dogs, small-breed dogs are considered geriatric at around 11 years of age, medium-breed dogs at 10 years, large-breed dogs at 8 years, and giant-breed dogs at 7 years (29). This breed-specific aging pattern was incorporated into the analysis to provide more accurate context for interpreting age-related variables.

In addition, to perform a more robust statistical analysis, comorbidities were classified into categories (e.g., cardiovascular, musculoskeletal, liver disease etc.) according to the system/organ involved (Supplementary Table A7).

### 2.6 Statistical analysis

The statistical analysis for this study was conducted using SPSS (IBM SPSS Statistics for Windows, Version 29.0.2.0, Armonk, NY: IBM Corp). Descriptive statistics were employed to summarize demographic and clinical characteristics, including frequencies, percentages, means, medians, and standard deviations. Normality of continuous variables was assessed using the Kolmogorov–Smirnov or Shapiro-Wik tests. For comparisons across groups, parametric and non-parametric tests were utilized based on data distribution. Specifically, chi-square tests were applied for categorical variables, while Kruskal-Wallis and Mann–Whitney U tests were used for non-parametric comparisons. To assess the difference in mean urine specific gravity (USG) across the four IRIS stages, a one-way analysis of variance (ANOVA) was performed, followed by post-hoc pairwise comparisons using t-tests with a Bonferroni correction. Pearson correlation and linear regression analyses were performed to evaluate relationships between continuous variables, and point-biserial correlation coefficients were calculated for binary and ordinal variable associations. Permutational Multivariate Analysis of Variance (PERMANOVA) was used to assess the impact of categorical variables on multidimensional VetMetrica™ scores. Odds ratios (OR) with 95% confidence intervals (CI) were calculated to evaluate the likelihood of QoL deterioration associated with specific comorbidities. The multiple linear regression analysis was conducted using software R (version 2024.04.1) to determine the relationship between the independent variables (comorbidities) and the deterioration in QoL scores for each domain. The significance of each predictor was assessed using t-tests, and the overall model fit was evaluated using R-squared and F-tests. The definition of deterioration in HRQL domain score is median HRQL domain score without concomitant conditions minus the HRQL domain score measured >0. All tests were two-tailed, and a significance level of *p* < 0.05 was considered statistically significant.

## 3 Results

### 3.1 Number of cases and participants

In this study, a total of 57 practitioners provided cCKD data on cases monitored in their clinics. Among them, the majority (49, 86.0%) self-identified as General Practitioners (GPs), while eight of the practitioners were referral clinicians. Approximately half of the data (106/215, 49.3%) was obtained from 12 major veterinary clinics in Athens and other cities, while the remaining half (109/215, 50.7%) was contributed by 45 practitioners, which represent 78.9% of the total participants. Each clinic provided on average 3.8 cases (range: 1–17). In total, 215 cCKD cases were collected in the period January 2023 – October 2023, of which the majority (n = 171, 79.6%) were IRIS Staged 1 and 2. A total of 20.5% (44/215) cases were IRIS Staged 3 and 4 in Time point 1. Timepoint 2 measurements were completed for 200 cases, as 12 dogs died in the 30-day interval between the two timepoints and three dogs were lost for various reasons in the follow-up period (owner moved out of town, refused second examination, etc.). The distribution of the recorded cases per IRIS Stage reported by the practitioners for Timepoints 1 and 2 is presented in detail in Table 1.

**Table 1:** Distribution of cCKD cases by IRIS stage as reported by the practitioners (Timepoint 1 and Timepoint 2).

IRIS Stages	Timepoint 1 *N* (%, 95CI)	Timepoint 2 *N* (%, 95CI)
Stage 1	53 (24.7, 19.0–31.0)	71 (35.5, 28.9–42.6)
Stage 2	118 (54.9, 48.0–61.7)	96 (48.0, 40.9–55.2)
Stage 3	29 (13.5, 9.2–18.8)	22 (11.0, 7.0–16.2)
Stage 4	15 (7.0, 4.0–11.3)	11 (5.5, 2.8–9.6)
Total	215 (100.0)	200 (100.0)

Among the 200 cases with data collected in both Timepoints 1 and 2, the reported IRIS stage remained consistent in 145 cases. However, in 55 cases, a change in IRIS Stage was reported; 20 (10%) cases were classed in a higher stage and 35 (17.5%) were classed in a lower stage. Further statistical analysis was performed using Timepoint 1 data IRIS Stage, corresponding to a larger sample size.

### 3.2 Canine demographics (gender, breed, weight, age)

Female (106/215, 49.3%) and male dogs (109/215, 50.7%) were equally represented. More female dogs have undergone ovariohysterectomy than males had undergone orchiectomy (chi-square = 29.5, df = 1, N = 215, *p* < 0.001). In females, a total of 85 cases were classified as Stage 1 and 2 (80.1%) and 21 cases as stages 3 and 4 (19.8%). For the male dogs the respective numbers were 86 (78.8%) and 23 (21.1%) (Table 2). No statistically significant correlation was identified between the sex and the IRIS Stage of the cases reported (chi-square = 6.4, df = 9, *N* = 215, *p* = 0.704).

**Table 2:** Sex and weight distribution of cCKD cases by IRIS stage.

Characteristic	Category	Stage 1, *N* (%)	Stage 2, *N* (%)	Stage 3, *N* (%)	Stage 4, *N* (%)	Total *N* (%)
Sex	Female intact	5 (9.4)	6 (5.1)	3 (10.4)	1 (6.7)	15 (7.0)
	Female spayed	21 (39.6)	53 (44.9)	10 (34.5)	7 (46.7)	91 (42.3)
	Male intact	11 (20.8)	27 (22.9)	11 (37.9)	4 (26.6)	53 (24.7)
	Male neutered	16 (30.2)	32 (27.1)	5 (17.2)	3 (20.0)	56 (26.0)
	Total by sex	53 (100.0)	118 (100.0)	29 (100.0)	15 (100.0)	215 (100.0)
Weight group	<6.75 Kgs	15 (28.3)	30 (25.4)	8 (27.6)	4 (26.7)	57 (26.5)
	6.75 - < 11.25 Kgs	9 (17.0)	37 (31.4)	4 (13.8)	4 (26.7)	54 (25.1)
	11.25 - < 20.25 Kgs	7 (13.2)	18 (15.3)	6 (20.7)	2 (13.3)	33 (15.3)
	20.25 - < 29.25 Kgs	11 (20.8)	14 (11.9)	7 (24.1)	3 (20.0)	35 (16.3)
	>29.25 Kgs	11 (20.8)	19 (16.1)	4 (13.8)	2 (13.3)	36 (16.7)
	Total by weight	53 (100.0)	118 (100.0)	29 (100.0)	15 (100.0)	215 (100.0)

Forty-six distinct breeds accounted for 62.3% (134/215) of the canine population, while mixed breeds made up 37.7% (81/215). Among the purebred dogs, the Maltese breed was the most common (18/215, 8.3%), followed by the Yorkshire Terriers (15/215, 6.9%) and the Labrador Retrievers (11/215, 5.1%) (Supplementary Table A8). Notably, 77.7% (63/81) of the mixed breed dogs and 80.6% (108/134) of the purebreds were classified as early IRIS stages (1 and 2) aligning with the overall study finding that approximately 80% of the study population was classified in these early stages. There was no statistically significant association between the dog breed and the IRIS stage reported (chi-square = 131.2, df = 141, *N* = 215, *p* = 0.712).

**Figure 1:**
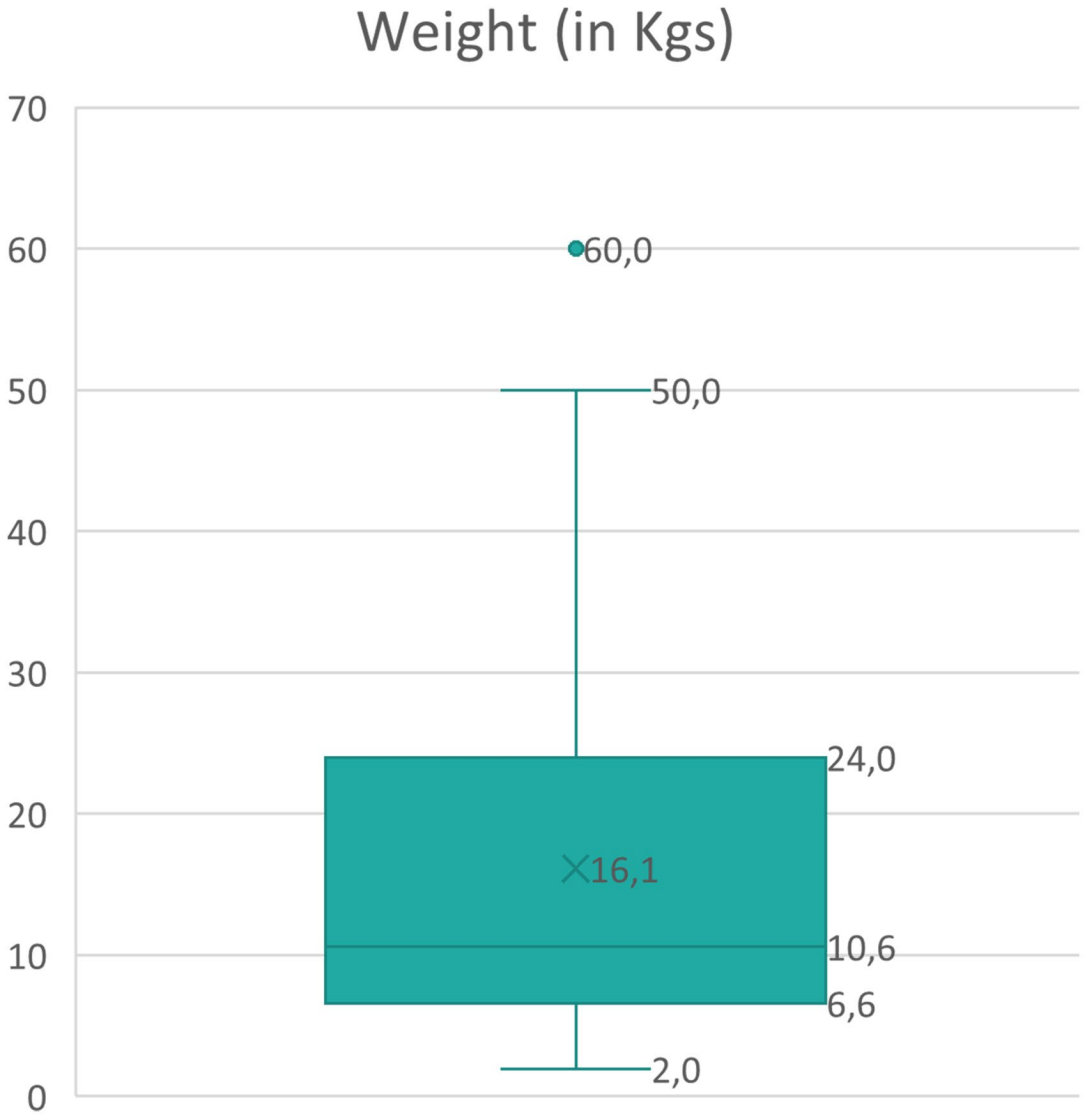
Boxplot of the canine weight reported in cCKD cases.

Regarding the weight of the dogs reported (K-S normality test = 0.181, *p* < 0.001, not normal distribution), the median weight of dogs was 10.6 kgs (SD = 11.9, range 1.9–60); approximately 50% of the cases were small-sized dogs (Figure 1). The total number of dogs in each weight group was distributed across the IRIS stages, as shown in Table 2. The analysis revealed no statistically significant difference among the weight groups and the reported IRIS stages (chi-square = 9.8, df = 12, *N* = 215, *p* = 0.635).

Regarding the canine age reported (K-S normality test = 0.076, *p* = 0.158, distribution can be considered reasonably consistent with a normal distribution, see Supplementary Figure A1), the mean age of the dogs was 10.7 years old (SD = 3.9). The minimum age was three years old (which also stands for the minimum age for a dog to participate in the study, according to the inclusion criteria), while the oldest dog was 18 years old. The mean age of dogs in Stages 1 to 4 was 9.8 (SD = 3.8), 11.3 (SD = 3.9), 9.6 (SD = 3.9) and 11.1 (SD = 3.6) respectively, not showing any significant correlation between age of reported cases and severity of the disease, as depicted by IRIS Stage.

**Figure 2:**
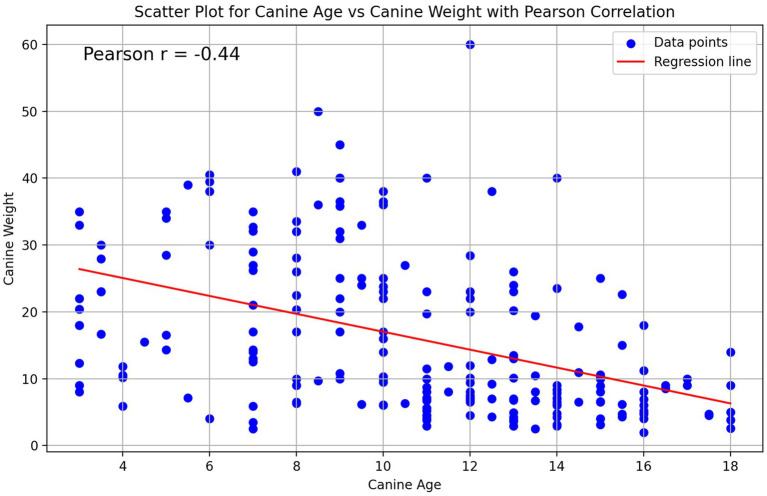
Scatter plot for canine age and weight with Pearson correlation.

Upon analysis of canine age across the defined weight groups, a statistically significant correlation was observed. In lower weight groups (corresponding to small-sized breeds), the mean age reported was 12.9 (<6.75 Kgs) and 12.0 (6.75 - < 11.25 Kgs) years, while in higher weight groups, the mean age decreased to as low as 9.0 (20,25 - < 29.25 Kgs) and 7.9 years old (>29.25 Kgs) (One Way Anova *F* = 17.8, df = 4, *p* < 0.001). This was also demonstrated by Pearson test (*r* = −0.44, p < 0.001) and linear regression analysis (estimated slope of −1.34, *p* < 0.001) (Figure 2). Furthermore, our results indicate that based on the UC Davis Book of Dogs, approximately one quarter of the cCKD cases reported, were not yet considered geriatric patients according to their weight group (29) (Supplementary Table A9).

### 3.3 Health-related quality of life measurement (VetMetrica™) and IRIS stages

To evaluate the impact of different stages of cCKD (IRIS Stages) on the quality of life (QoL) of dogs, owners were asked to complete the VetMetrica™ Canine HRQL assessment on the same day but independently of the clinical consultation. A total of 215 owner-reported HRQL assessments were recorded across each study phase, with only phase A considered for all calculations involving VetMetrica™ HRQL assessments.

Interestingly, a trend of deterioration in the QoL of dogs with cCKD was observed as the disease progressed. However, differences among the IRIS stages were not statistically significant (Supplementary Figure A2). When comparing Early (IRIS: 1 + 2) and Late (IRIS: 3 + 4) stages of cCKD, this trend of QoL deterioration was confirmed. The differences in the domain scores were statistically significant for Energetic/Enthusiastic domain (*p* = 0.013, Mann–Whitney tests) and the PWB domain (*p* = 0.023) (Table 3).

**Table 3:** Mann–Whitney *p*-values and medians in the various VetMetrica scores for early and late IRIS stages.

Domain	U stat	*p*-value	Stage 1–2 median	Stage 3–4 median
Energetic/Enthusiastic	4602.5	*0.013	35.91	27.52
Active/Comfortable	4047.0	0.340	34.69	32.86
Happy/Content	4363.0	0.069	36.88	33.75
Calm/Relaxed	3645.5	0.887	48.09	48.25
Physical Wellbeing	4528.5	*0.023	35.38	29.87
Emotional Wellbeing	4065.0	0.315	42.03	41.87

The statistically significant results are depicted with an asterisk.

### 3.4 Comorbidities

Most of the dogs (73.5%) presented with at least one comorbidity, while 26.5% (57 dogs) had no reported concomitant conditions. When diagnoses were grouped into broader disease categories, cardiovascular disease was the most prevalent, affecting 28.8% of the dogs. Other common categories included musculoskeletal issues (18.6%), liver disease (17.2%), and renal or urinary tract diseases (10.7%). The single most frequently reported individual diagnosis was periodontal disease, which was also present in 26.5% of dogs (Table 4). Other common specific conditions included degenerative joint disease/arthritis (39/215, 18.1%) and mild and moderate or severe liver disease (36/215, 16.7%) (Supplementary Table A10). Apart from the 26 concomitant diseases that were provided as a choice in the vet completion form, practitioners added other 14 in the “Other, please specify” text box (Supplementary Table A10).

**Table 4:** Frequency table of categories of concomitant diseases.

Category	*N*	%	95% CI
Cardiovascular	62	28.8	22.8–34.9
No concomitant conditions	57	26.5	20.6–32.4
Periodontal disease	57	26.5	20.6–32.4
Musculoskeletal	40	18.6	13.4–23.8
Liver	37	17.2	12.2–22.3
Renal disease/Urinary Tract	23	10.7	6.6–14.8
Hematologic	20	9.3	5.4–13.2
Dermatologic	19	8,8	5.0–12.6
Obesity	11	4.7	2.2–8.1
Endocrine/metabolic	10	4.7	1.8–7.5
Pathogenic infection	9	4.2	1.5–6.9
Nervous system	8	3.7	1.2–6.3
Cancer	6	2.8	0.6–5.0
GI disease	6	2.8	0.6–5.0
Other	8	3.7	1.2–6.3

**Figure 3:**
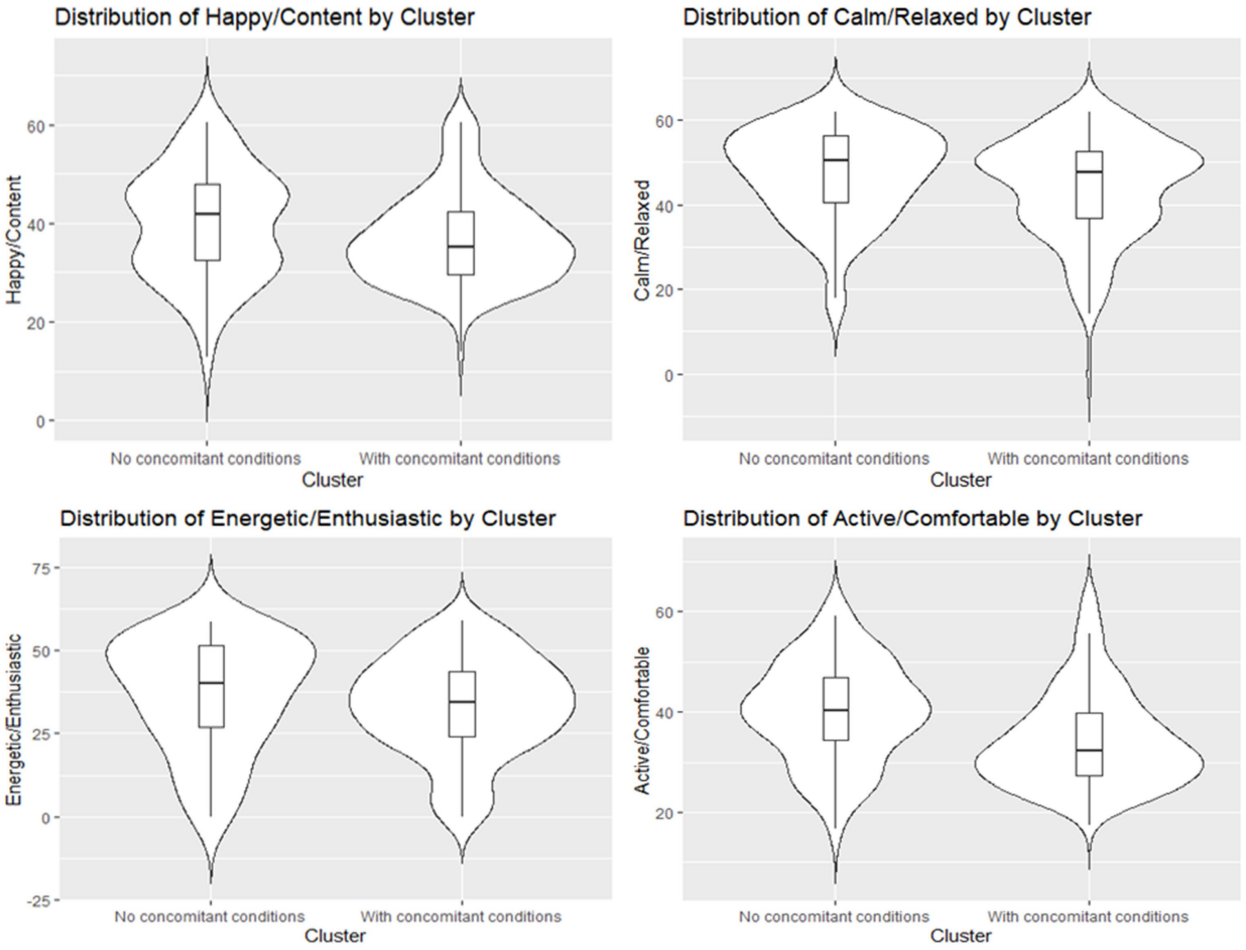
Violin plots with an overlaid boxplot of the four VetMetrica domain scores across the two groups (Dogs with cCKD and no comorbidities vs. Dogs with cCKD and comorbidities).

To address whether the presence of comorbidities affect the overall HRQL in dogs with cCKD, a Permutational Multivariate Analysis of Variance (PERMANOVA) and Kruskall-Wallis test was performed; the results indicate that, at a 5% significance level, the group variable has a statistically significant effect on the four-dimensional vector of Domain scores [*F*(1, 213) = 4.6936, *p*-value = 0.019 < 0.05] (Figure 3). Moreover, Kruskal-Wallis results indicate that there is no statistically significant difference in the Energetic/Enthusiastic scores across the two groups (chi-squared = 3.1758, df = 1, *p*-value = 0.07474) and in the Calm/Relaxed scores between the two groups (chi-squared = 3.0153, df = 1, *p*-value = 0.08248). On the other hand, there is a statistically significant difference in the Active/Comfortable scores between the groups (chi-squared = 16.02, df = 1, *p*-value = 6.266e-05) and in the Happy/Content scores across the two groups (chi-squared = 3.978, df = 1, *p*-value = 0.0461), with significantly higher VetMetrica scores in dogs with cCKD and no comorbidities compared to dogs with cCKD and with comorbidities.

The Odds Ratio analysis performed, showed statistically significant associations between Cardiovascular, Liver, Musculoskeletal and Periodontal comorbidities and a higher likelihood of deterioration in specific HRQL domains (Table 5). More specifically, the Active/Comfortable domain was the most frequently affected, with significantly higher odds of deterioration in dogs with musculoskeletal (OR 6.6, *p* < 0.001), liver (OR 4.2, *p* = 0.005), cardiovascular (OR 3.3, *p* = 0.002), or periodontal disease (OR 2.4, *p* = 0.019). Similarly, the odds of deterioration in the Energetic/Enthusiastic domain were significantly increased by musculoskeletal (OR 3.7, *p* = 0.002), liver (OR 2.7, *p* = 0.017), and cardiovascular (OR 2.3, *p* = 0.013) comorbidities. Deterioration in the Happy/Content domain was significantly associated with liver (OR 2.9, *p* = 0.021) and musculoskeletal (OR 2.7, *p* = 0.025) diseases. Notably, only liver disease was significantly linked to a deterioration in the Calm/Relaxed domain (OR 4.3, *p* = 0.001).

**Table 5:** Odds Ratios for VetMetrica HRQL domain deterioration by comorbidity category in dogs with cCKD.

Category of comorbidities	Domain SCORE	OR	95%CI	*p*-value
Cardiovascular	Energetic/Enthusiastic	2.3	1.182–4.401	*0.0134
Cardiovascular	Happy/Content	1.979	1.021–3.928	0.0543
Cardiovascular	Active/Comfortable	3.305	1.569–7.299	*0.0017
Cardiovascular	Calm/Relaxed	1.252	0.6833–2.292	0.541
Liver	Energetic/Enthusiastic	2.701	1.227–5.850	*0.0169
Liver	Happy/Content	2.901	1.178–6.966	*0.0208
Liver	Active/Comfortable	4.195	1.526–11.41	*0.0052
Liver	Calm/Relaxed	4.314	1.767–10.29	*0.0008
Musculoskeletal	Energetic/Enthusiastic	3.704	1.633–9.319	*0.002
Musculoskeletal	Happy/Content	2.652	1.160–6.717	*0.0254
Musculoskeletal	Active/Comfortable	6.599	2.085–21.00	*0.0004
Musculoskeletal	Calm/Relaxed	2.071	0.9858–4.568	0.074
Periodontal disease	Energetic/Enthusiastic	1.936	1.028–3.678	0.0574
Periodontal disease	Happy/Content	1.91	0.9557–3.826	0.0719
Periodontal disease	Active/Comfortable	2.44	1.188–4.952	*0.0187
Periodontal disease	Calm/Relaxed	1.668	0.6636–4.268	0.3706

The statistically significant results are depicted with an asterisk.

The Odds Ratio analysis for the PWB (Physical Wellbeing) and EWB (Emotional Wellbeing) domains revealed similar results (Table 6). The strongest association for the PWB was with liver disease (OR 5.1, *p* = 0.001) and musculoskeletal disease (OR 4.3, p = 0.002), followed by renal/urinary tract disease (OR 3.7, *p* = 0.034), cardiovascular disease (OR 2.1, *p* = 0.038), and periodontal disease (OR 2.0, *p* = 0.050). For EWB summary score, only the presence of liver (OR 3.0, *p* = 0.010) and musculoskeletal disease (OR 2.1, *p* = 0.052: not reached the conventional threshold for statistical significance but suggests a trend that may warrant further investigation with a larger sample size) was associated with a higher likelihood of deterioration.

**Table 6:** Odds ratios analysis for the summary physical wellbeing (PWB) and emotional wellbeing (EWB) domains deterioration by comorbidity category in dogs with cCKD.

Disease	Score	OR	95%CI	*p*-value
Cardiovascular	PWB	2.093	1.083 to 4.146	*0.0379
Cardiovascular	EWB	1.286	0.7023 to 2.353	0.4477
Liver	PWB	5.1	1.866 to 13.84	*0.001
Liver	EWB	3.027	1.287 to 6.547	*0.0096
Musculoskeletal	PWB	4.343	1.656 to 10.61	*0.0015
Musculoskeletal	EWB	2.12	1.009 to 4.674	*0.052
Periodontal disease	PWB	2.017	1.012 to 4.045	*0.0506
Periodontal disease	EWB	1.06	0.5643 to 1.971	0.8767
Renal disease/Urinary tract	PWB	3.74	1.177 to 12.22	*0.0341
Renal disease/Urinary tract	EWB	2.157	0.8258 to 5.542	0.1242

The statistically significant results are depicted with an asterisk.

Finally, multiple regression analysis, which examines the combined impact of various comorbidities on the HRQL domain scores of dogs with cCKD, reveals that the presence of musculoskeletal and liver diseases as concomitant conditions significantly exacerbates the deterioration in HRQL within the indicated domains. This deterioration is attributable to the presence of these comorbidities, independent of other variables. Specifically, dogs with cCKD associated with musculoskeletal diseases as comorbidity were found to experience a deterioration of 6.24 in the Energetic/Enthusiastic domain score, 4.09 for Happy/Content, 6.47 for Active /Comfortable, and 6.35 for Physical Wellbeing. In addition, dogs with cCKD associated with Liver diseases as comorbidity were found to experience a deterioration of 4.81 in the Active /Comfortable domain score (Table 7).

**Table 7:** Statistically significant results of multiple regression analysis for the combined impact that the comorbidities have on each of the HRQL domain scores of dogs with cCKD.

Comorbidity	Domain score	Expected domain score when no comorbidity	Deterioration in the domain score	SD±	*p*-value
Musculoskeletal	Energetic/Enthusiastic	37.62 (±1.62)	6.24	2.88	0.0316
	Happy/Content	40.27 (±1.03)	4.09	1.83	0.0267
	Active/Comfortable	40.03 (±0.96)	6.47	1.71	0.0002
Liver	Active/Comfortable		4.81	1.75	0.007
Musculoskeletal	Physical Wellbeing	38.82 (±1.21)	6.35	2.16	0.0036

### 3.5 Clinical signs

The most prevalent clinical signs reported in dogs with cCKD were polyuria (110/215, 51.2%) and halitosis (110/215, 51.2%), followed by polydipsia (107/215, 49.8%), weakness (88/215, 40.9%), anorexia (75/215, 34.9%), weight loss (60/215, 27.9%) and vomiting (57/215, 26.5%). All reported signs are included in the Supplementary Table A11.

To evaluate possible correlation between the presence of a clinical sign (binary variable) and the severity of cCKD as depicted by the IRIS Stage reported (considered for this analysis as an ordinal variable), point-biserial correlation coefficient was calculated (*r_pb_*). The coefficient can range from −1 to +1, a value close to +1 indicating a strong positive association, meaning that the presence of a symptom is associated with a higher IRIS Stage, while a value near −1 indicates a strong negative association, meaning that a symptom is associated with a lower IRIS Stage. A positive correlation was found for all the clinical signs, indicating that their frequency increased with advancing IRIS stage. This association was statistically significant (*p* < 0.05) for polydipsia, polyuria, weight loss, anorexia, vomiting, diarrhea, halitosis, pale gums and gastroenteritis (Supplementary Table A12).

Owners were asked which of the reported clinical signs they considered more troublesome for their dog as well as for themselves. Interestingly, in 56/215 cases (26.1%) the owners did not declare any symptom as being troublesome for them or the dog. In the rest of the cases, and taking into account the 95% confidence intervals, the clinical signs reported as more troublesome for the dog were diarrhea, vomiting, fragile bones, problems with vision and weakness/fatigue. Similarly, halitosis, vomiting, polyuria, diarrhea and anorexia were reported as more troublesome for the owner. Halitosis was reported as significantly more troublesome for the owner than the dog and weakness and problems with vision were reported as significantly more troublesome for the dog (Supplementary Table A13).

### 3.6 Disease history and diet

Practitioners were asked to comment in an open text box whatever they thought was important regarding the case history of the dog included in the study. In a 25.6% of cases, cCKD was diagnosed due to the clinical signs exhibited by the dogs, which prompted their presentation to the veterinarian (55/215, 25.6%). Notably, 24.2% of dogs (52/215) were reported to have a previous history of Canine Leishmaniosis (CanL). In 29 cases (13.5%) the practitioners reported that cCKD was diagnosed during routine check-ups. Finally, in 24 cases (11.2%) history of various infectious and non-infectious diseases were reported (i.e., Ehrlichiosis, Cushing’s disease, urinary tract infections, cardiovascular diseases etc.)

Regarding diet, a significant proportion of the dogs (136/215, 63.3%) were prescribed a clinical renal diet and 27.5% (59/215) of the dogs were provided with home-cooked nutrition. Commercial dry food was provided to 49/215 cases (22.8%) and other types of food such as commercial canned food and raw food were reported in smaller percentages (7.5 and 3.3% respectively).

### 3.7 Biochemical examinations and urinalysis

The study population showed biochemical abnormalities consistent with cCKD. The descriptive statistics for all biochemical parameters are presented in Table A14. Key indicators of renal function were elevated, with a mean bCREA of 2.28 ± 1.49 mg/dL and a mean BUN of 59.10 ± 46.76 mg/dL. The mean P concentration was also elevated at 6.22 ± 3.57 mg/dL. In contrast, the mean ALB concentration was low at 2.77 ± 0.48 g/dL. Electrolyte analysis revealed a mean chloride level of 107.78 ± 5.59 mmol/L, which is above the reference range, while other electrolytes, including sodium, potassium, and calcium, were within reference range.

Urinalysis findings were consistent with the biochemical evidence of reduced renal function. The mean USG for the entire population was 1020.89 ± 9.39. A statistically significant difference in the mean USG among the four IRIS stages was found using ANOVA (*F* = 12.51, *p* < 0.001). The pairwise comparisons using independent samples *t*-test, showed that the mean USG was significantly different between all IRIS stages (*p* = 0.005) except for the mean USG between Stage 3 and Stage 4 (*p* = 0.417).

### 3.8 Owners demographics

Almost two thirds of the participating dog owners were females (140/215, 65.1%) and one third of them males (75/215, 34.9%). Both sexes exhibited similar mean age and age distribution, as mean age in women was 47.5 years old and mean age in men was 45.9 years old. Analytical demographics regarding age, employment and education status are included in the Supplementary Table A15.

## 4 Discussion

This observational study provides real-world data on cCKD from Greece, contributing to the existing literature by revealing the following critical insights. First, approximately 80% of the reported cCKD cases were classified at early, manageable IRIS stages providing a window for intervention by the responsible veterinarian. Second, comorbidities were not only concurrent conditions, but key drivers of QoL deterioration, even in early IRIS stages. Lastly, larger-sized dogs were reported as cCKD cases by veterinarians at younger ages compared to smaller-sized dogs. Notably, approximately one quarter of all reported cCKD cases fall below the geriatric age for their weight group, indicating that cCKD concerns young dogs as well.

The high prevalence of cCKD cases classified as IRIS stages 1 and 2 in our study aligns with previous studies (4, 12) where the early IRIS Stages 1 and 2 were reported more frequently (77.7 and 78.6% respectively) but contrasts with the findings of a retrospective study conducted over a 10-year period, where most cases (75.7%) were classified as IRIS stages 3 and 4 (30). The distribution of IRIS stages in our study indicates that many dogs were diagnosed and managed in earlier stages of cCKD, which is favorable for prognosis and QoL of both the animal and the owner. This may be attributed to the increased awareness among Greek veterinarians. A contributing factor is likely the endemic nature of CanL in Greece (31). Given the strong association between CanL and cCKD and the current guidelines on CanL diagnosis and management in dogs, veterinarians often include biochemical examinations to assess renal function alongside serological tests for anti-*Leishmania* IgG antibodies (32). As a result, the early detection of cCKD becomes more likely, even before clinical signs become apparent. Notably, in our study 24.2% of dogs had a previous history of CanL. This becomes nowadays particularly relevant beyond the geographical limits of Greece and other endemic countries, as CanL is now evidenced in previously unaffected countries due to climate change, increased international travel and pet adoptions from endemic areas, suggesting that veterinarians in other countries should also consider CanL as a potential underlying cause of cCKD (33–35).

A primary finding of this study is the negative impact of comorbidities on the QoL of dogs with cCKD. Dogs with no comorbidities had significantly higher QoL scores than those with concomitant diseases. The most substantial impact was observed with Degenerative Joint Disease/Arthritis (present in 18.1% of the study population) which was associated with an approximately seven-fold greater likelihood of QoL deterioration. This highlights the need to treat pain associated with Degenerative Joint Disease/Arthritis to improve the QoL of the animal, even in early IRIS stages. While the use of NSAIDs presents a challenge due to potential renal adverse effects (36), established guidelines allow their safe administration (37). Similarly, dogs with concurrent liver disease (present in 15.8% of cases) and cardiovascular diseases (including heart murmurs in 13.0% and diagnosed mitral valve disease in 9.3%) were approximately three and four times more likely to experience QoL deterioration. Periodontal disease - the most common comorbidity in our study at 26.5% - was associated with an approximately twofold deterioration in PWB. This high prevalence was anticipated as periodontitis is a frequently diagnosed condition in veterinary practice (38). Moreover, there is an increased likelihood of developing periodontitis as age increases and bodyweight decreases (39) and previous studies have demonstrated an association between periodontitis and pathological changes in the kidneys, myocardium, and liver of dogs (40). Collectively, these results demonstrate the necessity of a holistic approach to managing dogs with cCKD. The early diagnosis and management of concurrent conditions are critical to improving the overall well-being and QoL of these animals.

Beyond the burden of comorbidities, the progression of cCKD itself, as reflected by IRIS staging, also emerged as a determinant of patient QoL. Late-stage cCKD (IRIS stages 3 & 4) were directly linked to worsened QoL of the animal. This was most pronounced in Physical Well Being domain but was also evident in the Energetic/Enthusiasm, Happiness/Contentment, and Activity/Comfort domain scores. As cCKD progresses, it affects QoL of the canine patient, as it is perceived by the pet owner, thus highlighting the importance for early diagnosis and timely intervention. Importantly, although the differences in HRQL across different IRIS stages were not statistically significant, a trend of deterioration in QoL was observed for E/E, H/C, and A/C domains. However, this was not the case for C/R domain, probably because it is affected by the temperament of the dog. The reduced number of animals in the late stages may have contributed to the lack of statistical significance when considering all IRIS stages. However, the impact of disease progression became clear when the stages were grouped: dogs in late stages (3 and 4) had statistically significant lower scores for both Energetic/Enthusiasm and overall Physical Wellbeing compared to those in early stages (1 and 2). Disease progression was also associated with increased frequency of serious clinical signs, like polydipsia, polyuria, weight loss, anorexia, vomiting, diarrhea, halitosis etc. which also aligns to previous studies (1, 41). Reporting clinical signs by the owners such as polydipsia and polyuria is strongly supported by the urinalysis data from our study. We found a statistically significant decrease in mean USG as the IRIS stage advanced which aligns with previous studies (42). This demonstrates the progressive loss of renal concentrating ability, a critical element of cCKD. Interestingly, while the decline in USG was significant across the early stages, the difference between IRIS stage 3 and stage 4 was no longer significant, suggesting that urine concentrating ability is already severely compromised by the time a dog reaches stage 3 and does not change dramatically when progressing to the final stage. This shows the importance of proactive management to slow disease progression, even in early stages (1 and 2) when animals show absent to mild clinical signs. This does not only protect the animal’s health but has a direct impact on the QoL as perceived by the owner. Many clinical signs that are troublesome for the pet owner and the dog increase in frequency with advancing IRIS stage. Therefore, initiating management in the early cCKD stages will have a valuable positive impact to protecting the QoL of both the dog and the owner.

In our study larger-breed dogs were reported as cCKD cases at significantly younger ages (8–9 years old) than their smaller – breed counterparts (12–13 years old), a finding that aligns with their shorter general longevity patterns (29). This finding suggests that vigilance is required in younger, large-breed dogs and that breed-specific screening protocols should be considered. Veterinarians should not wait for large-breed dogs to reach the “geriatric” age before beginning renal health monitoring. Moreover, while geriatric age occurs later in small and medium-sized breeds compared to large and giant breeds, it is noteworthy that, in our study, 25–30% of the reported cases fall below the geriatric age threshold for each weight group, highlighting that cCKD is not exclusively a geriatric issue. While the mean age of cCKD cases (10.7 years) is consistent with previous studies showing that cCKD is primarily a disease of older dogs (43, 44), the lack of significant correlation between age and IRIS stage suggests that age alone does not predict disease severity. Our data also show that sex, breed and weight group did not correlate with disease severity, suggesting that cCKD progresses similarly across these demographics once established.

Our findings also highlight the inherent challenges of IRIS staging in a real-world general practice setting, especially in early phases of the disease. The observed shifts in IRIS stages between two timepoints within a 30-day interval - with 10% of dogs being classified at a higher IRIS stage and 17.5% being classified at a lower IRIS stage - do not necessarily indicate disease progression or regression. Besides, the latter is unlikely given that kidney failure in cCKD is generally irreversible (45). While acute episodes during cCKD can lead to transient elevations in bCREA (46) that may influence staging, such alterations are most probably attributed to daily intra-individual variations in bCREA, an inherent limitation of IRIS staging system.

When interpreting the results of this study, several limitations must be taken into account. A primary consideration is the study’s geographical scope, which was confined to Greece. This restricts the direct generalizability of the findings to other regions with different environmental factors, genetic pools, animal healthcare systems and the epidemiological landscape, particularly concerning infectious and parasitic diseases that may be associated with renal function such as CanL. Moreover, the sample size of 215 cases, while adequate for the conclusions drawn, may limit the detection of subtle disease patterns in specific breeds. In the future, larger multicenter studies should address this issue and improve statistical power. The underrepresentation of advanced cases (IRIS Stages 3 and 4) also means that while our findings on early-stage disease are robust, the full spectrum of late-stage cCKD may not be completely described. However, given the scarcity of real-life data on cCKD in this area, this study could potentially serve as a foundation for future research.

As for the limitations in data collection, the reliance on owner-reported information introduces a potential for recall bias and subjective interpretation of clinical signs, although this was mitigated through the use of standardized questionnaires with multiple choice options to enhance the consistency and reliability of the data. Additionally, SDMA, a key biomarker for early detection, and blood pressure measurements, which are important as hypertension can impact both QoL and disease progression (47), were not included in this study’s protocol. Likewise, Body Condition Score (BCS) and Muscle Condition Score (MCS), which are also important indicators of patient status and QoL, were not recorded during the study period. Their absence limited our ability to assess the potential influence of nutritional or muscular status on disease progression and QoL (48).

Although these limitations may have impacted the precision of IRIS staging, enzymatic creatinine-based IRIS staging is a widely accepted clinical standard, still used by most practitioners in daily practice and has been considered sufficient for the study’s objectives and to reflect the real-world scenario and its difficulties. Indeed, this observational study highlighted the diagnostic challenges that practitioners face when staging cCKD. The apparent disease regression based on the IRIS stage in some cases, particularly the transition from IRIS stage 2 to stage 1 in 23 cases, illustrated the difficulties in accurately staging at the early phases of the disease. It emphasized the importance of careful staging and monitoring over time to diagnose and manage cCKD, while also stressing the need for general practitioners to receive reliable guidance, support, and specialized training from experts. Future research should address these limitations by incorporating longer follow-up periods, larger sample sizes, and additional biomarkers, which would enhance the understanding and management of cCKD in different areas.

## 5 Conclusion

This study provides valuable RWD on cCKD cases in Greece, contributing to the global understanding of this important health condition. The findings demonstrate the importance of early diagnosis and comprehensive management, as most cases were identified in early IRIS stages (1 and 2), which are associated with better prognosis and QoL outcomes. The study highlights that cCKD is not exclusively a geriatric issue, with a significant proportion of cases observed in dogs below the geriatric age threshold, particularly in larger breeds.

The QoL of dogs with cCKD deteriorates as the disease progresses, and this is worsened by the presence of comorbidities, especially degenerative joint disease, liver disease cardiovascular conditions, periodontal disease, and renal/urinary tract diseases, even in early stages of cCKD. This highlights the need for early and integrated management strategies that address both cCKD and its associated conditions, in ways that do not further impact kidney function. Additionally, the increase in IRIS stage was linked to higher prevalence of clinical signs, such as polyuria, anorexia, vomiting, and halitosis, which were reported as troublesome by the owners for themselves and/or their dog.

The study also highlights the value of using validated tools such as the VetMetrica™ instrument, to assess HRQL in dogs, providing a more comprehensive understanding of the impact that diseases have to the animal’s well-being. Furthermore, the high prevalence of previous history of CanL among the study population suggests that veterinarians in non-endemic areas should remain vigilant about this potential underlying cause of cCKD, especially given the increasing spread of *Leishmania* spp. due to climate change and international pet travel.

Despite its limitations, including a focus on a single country and potential underrepresentation of advanced cCKD stages, this research lays the groundwork for future studies to expand the knowledge and evidence on this important disease. Studies incorporating additional biomarkers and longer follow-up periods will further refine diagnostic and management strategies. This study demonstrates the critical need for early detection, timely intervention, and holistic care to improve outcomes for both dogs with cCKD and their owners.

## Data Availability

The raw data supporting the conclusions of this article will be made available by the authors, without undue reservation.

## References

[1] O’Neill DG Elliott J Church DB McGreevy PD Thomson PC Brodbelt DC. Chronic kidney disease in dogs in UK veterinary practices: prevalence, risk factors, and survival. J Vet Intern Med. (2013) 27:814–21. doi: 10.1111/jvim.12090 23647231

[2] Pedrinelli V Lima DM Duarte CN Teixeira FA Porsani M Zarif C. Nutritional and laboratory parameters affect the survival of dogs with chronic kidney disease. Plos One. (2020) 15:e0234712. doi: 10.1371/journal.pone.0234712, PMID: 32603378 PMC7326232

[3] Lingaas F Tengvall K Jansen JH Pelander L Hurst MH Meuwissen T. Bayesian mixed model analysis uncovered 21 risk loci for chronic kidney disease in boxer dogs. Plos Genet. (2023) 19:e1010599. doi: 10.1371/journal.pgen.1010599, PMID: 36693108 PMC9897549

[4] Perini-Perera S Del-Ángel-Caraza J Pérez-Sánchez AP Quijano-Hernández IA Recillas-Morales S. Evaluation of chronic kidney disease progression in dogs with therapeutic Management of Risk Factors. Front Vet Sci. (2021) 8:621084. doi: 10.3389/fvets.2021.621084, PMID: 34026884 PMC8131674

[5] Sosnar M Kohout P Růžička M Vrbasová L. Retrospective study of renal failure in dogs and cats admitted to University of Veterinary and Pharmaceutical Sciences, Brno during 1999-2001. Acta Vet Brno. (2003) 72:593–8. doi: 10.2754/avb200372040593

[6] Kokkinos Y Morrison J Bradley R Panagiotakos T Ogeer J Chew D. An early prediction model for canine chronic kidney disease based on routine clinical laboratory tests. Sci Rep. (2022) 12:14489. doi: 10.1038/s41598-022-18793-6, PMID: 36008537 PMC9411602

[7] Vinodhini J Abiramy Prabavathy A Uma S Barathiraja S Rajkumar K Thanislass J. Clinico-biochemical findings associated with stage III and stage IV of chronic kidney disease in dogs. Int J Vet Sci Res. (2021) 7:156–62. doi: 10.17352/ijvsr.000096

[8] Spitznagel MB Jacobson DM Cox MD Carlson MD. Caregiver burden in owners of a sick companion animal: a cross-sectional observational study. Vet Rec. (2017) 181:321–1. doi: 10.1136/vr.104295, PMID: 28870976

[9] Jennings LB. Potential benefits of pet ownership in health promotion. J Holist Nurs. (1997) 15:358–72. doi: 10.1177/089801019701500404, PMID: 9397745

[10] McConnell AR Brown CM Shoda TM Stayton LE Martin CE. Friends with benefits: on the positive consequences of pet ownership. J Pers Soc Psychol. (2011) 101:1239–52. doi: 10.1037/a0024506, PMID: 21728449

[11] Cherniack EP Cherniack AR. Assessing the benefits and risks of owning a pet. Can Med Assoc J. (2015) 187:715–6. doi: 10.1503/cmaj.150274, PMID: 26078462 PMC4500685

[12] Wright AK Taylor D Lowe M Barlow S Jackson J. Replicating the real-world evidence methods available in human health to assess burden and outcomes for dogs with chronic kidney disease, their owners, and the veterinary healthcare system in the United States of America. Front Vet Sci. (2025) 12:12. doi: 10.3389/fvets.2025.1502933, PMID: 40061906 PMC11886590

[13] Hawkins RD Ellis A Robinson C. Exploring the connection between pet attachment and owner mental health: the roles of owner-pet compatibility, perceived pet welfare, and Behavioral issues. Psychiatry Clinical Psychol. (2024). doi: 10.1101/2024.11.20.24317636 PMC12520413 41086231

[14] Liu M Qi Y Wang W Sun X. Toward a better understanding about real-world evidence. Eur J Hosp Pharm. (2022) 29:8–11. doi: 10.1136/ejhpharm-2021-003081, PMID: 34857642 PMC8717805

[15] Ramamoorthy A Huang S. What does it take to transform real-world data into real-world evidence? Clin Pharmacol Ther. (2019) 106:10–8. doi: 10.1002/cpt.1486, PMID: 31273768

[16] Santos-Araújo C Mendonça L Carvalho DS Bernardo F Pardal M Couceiro J. Twenty years of real-world data to estimate chronic kidney disease prevalence and staging in an unselected population. Clin Kidney J. (2023) 16:111–24. doi: 10.1093/ckj/sfac206, PMID: 36726443 PMC9871850

[17] Li J An J Huang M Zhou M Montez-Rath ME Niu F. Representation of real-world adults with chronic kidney disease in clinical trials supporting blood pressure treatment targets. J Am Heart Assoc. (2024) 13:e031742. doi: 10.1161/JAHA.123.031742, PMID: 38533947 PMC11179783

[18] Rottura M Drago SFA Gianguzzo VM Molonia A Pallio G Scoglio R. Chronic kidney disease progression in diabetic patients: real world data in general practice. Heliyon. (2024) 10:e30787. doi: 10.1016/j.heliyon.2024.e30787, PMID: 38765038 PMC11096917

[19] Davies V Reid J Wiseman-Orr ML Scott EM. Optimising outputs from a validated online instrument to measure health-related quality of life (HRQL) in dogs. Plos One. (2019) 14:e0221869. doi: 10.1371/journal.pone.0221869 31532799 PMC6750605

[20] Reid J Wiseman-Orr L Scott M. Shortening of an existing generic online health-related quality of life instrument for dogs. J Small Anim Pract. (2018) 59:334–42. doi: 10.1111/jsap.12772, PMID: 29023735

[21] Armitage AJ Miller JM Sparks TH Georgiou AE Reid J. Efficacy of autologous mesenchymal stromal cell treatment for chronic degenerative musculoskeletal conditions in dogs: a retrospective study. Front Vet Sci. (2023) 9:1014687. doi: 10.3389/fvets.2022.1014687, PMID: 36713862 PMC9880336

[22] Reid J Gildea E Davies V Thompson J Scott M. Measuring the effect of the anti-nerve growth factor antibodies bedinvetmab and frunevetmab on quality of life in dogs and cats with osteoarthritis using a validated health-related quality of life outcome measure: an observational real-world study. Front Vet Sci. (2024) 11:1395360. doi: 10.3389/fvets.2024.1395360, PMID: 39205806 PMC11349630

[23] Valiakos G Pavlidou E Zafeiridis C Tsokana CN Del Rio Vilas VJ. Antimicrobial practices among small animal veterinarians in Greece: a survey. One Health Outlook. (2020) 2:7. doi: 10.1186/s42522-020-00013-8, PMID: 33829129 PMC7993541

[24] Vlachos NA Michail C Sotiropoulou D. Is ISO/IEC 17025 accreditation a benefit or hindrance to testing laboratories? The Greek experience. J Food Compos Anal. (2002) 15:749–57.

[25] Taverniers I Van Bockstaele E De Loose M. Analytical method validation and quality assurance In: Gad SC, editor. Pharmaceutical sciences Encyclopedia. 1st ed: Wiley (2010). 1–48.

[26] Greenberg N Roberts WL Bachmann LM Wright EC Dalton RN Zakowski JJ. Specificity characteristics of 7 commercial creatinine measurement procedures by enzymatic and Jaffe method principles. Clin Chem. (2012) 58:391–401. doi: 10.1373/clinchem.2011.172288 22166253

[27] Cobbaert CM Baadenhuijsen H Weykamp CW. Prime time for enzymatic creatinine methods in Pediatrics. Clin Chem. (2009) 55:549–58. doi: 10.1373/clinchem.2008.116863 19168555

[28] Hart BL Hart LA Thigpen AP Willits NH. Assisting decision-making on age of neutering for mixed breed dogs of five weight categories: associated joint disorders and cancers. Front Vet Sci. (2020) 7:472. doi: 10.3389/fvets.2020.00472, PMID: 32851043 PMC7412743

[29] Siegal M Barlough JE. University of California, Davis, editors. UC Davis book of dogs: The complete medical reference guide for dogs and puppies. 1st ed. New York: HarperCollins Publishers (1995). 538 p.

[30] Guidi G Rossini C Cinelli C Meucci V Lippi I. Canine chronic kidney disease: Retrospective study of a 10-year period of clinical activity In: Pugliese A Gaiti A Boiti C, editors. Veterinary science. Berlin, Heidelberg: Springer Berlin Heidelberg (2012). 115–8.

[31] Symeonidou I Sioutas G Gelasakis AI Tsokana CN Papadopoulos E. Leishmaniosis in Greece: the veterinary perspective. Pathogens. (2023) 12:769. doi: 10.3390/pathogens12060769, PMID: 37375459 PMC10303642

[32] LeishVet. Canine leishmaniosis: a practical guide for veterinarians. Available online at: https://www.leishvet.org/wp-content/uploads/2024/04/FS-ALIVE24-canine.pdf

[33] Roura X Cortadellas O Day MJ Benali SL Canine Leishmaniosis Working Group Zatelli A. Canine leishmaniosis and kidney disease: Q&a for an overall management in clinical practice. J Small Anim Pract. (2021) 62:E1–E19. doi: 10.1111/jsap.13237, PMID: 33107613

[34] Rocha R Pereira A Maia C. A global perspective on non-autochthonous canine and feline Leishmania infection and leishmaniosis in the 21st century. Acta Trop. (2023) 237:106710. doi: 10.1016/j.actatropica.2022.106710, PMID: 36198329

[35] Maia C Cardoso L. Spread of Leishmania infantum in Europe with dog travelling. Vet Parasitol. (2015) 213:2–11. doi: 10.1016/j.vetpar.2015.05.003, PMID: 26021526

[36] KuKanich B Bidgood T Knesl O. Clinical pharmacology of nonsteroidal anti-inflammatory drugs in dogs. Vet Anaesth Analg. (2012) 39:69–90. doi: 10.1111/j.1467-2995.2011.00675.x, PMID: 22151877

[37] Innes JF Clayton J Lascelles BD. Review of the safety and efficacy of long-term NSAID use in the treatment of canine osteoarthritis. Vet Rec. (2010) 166:226–30. doi: 10.1136/vr.c97, PMID: 20173106

[38] Niemiec BA. Periodontal disease. Top Companion Anim Med. (2008) 23:72–80. doi: 10.1053/j.tcam.2008.02.003 18482707

[39] Anderson KL Zulch H O'Neill DG Meeson RL Collins LM. Risk factors for canine osteoarthritis and its predisposing Arthropathies: a systematic review. Front Vet Sci. (2020) 7:220. doi: 10.3389/fvets.2020.00220, PMID: 32411739 PMC7198754

[40] Nabi SU Wani AR Shah OS Dey S. Association of periodontitis and chronic kidney disease in dogs. Vet World. (2014) 7:403–7. doi: 10.14202/vetworld.2014.403-407

[41] Oburai LN Vaikunta Rao V Naik BR. Clinical and nephrosonographic findings in canine chronic renal failure: a prospective study. Figshare. (2015) 8:11–16. doi: 10.9790/2380-08621116

[42] Kim J Lee CM Kim HJ. Biomarkers for chronic kidney disease in dogs: a comparison study. J Vet Med Sci. (2020) 82:1130–7. doi: 10.1292/jvms.20-0125, PMID: 32581150 PMC7468053

[43] Cianciolo RE Benali SL Aresu L. Aging in the canine kidney. Vet Pathol. (2016) 53:299–308. doi: 10.1177/0300985815612153 26508694

[44] Smets PMY Meyer E Maddens BEJ Duchateau L Daminet S. Urinary markers in healthy young and aged dogs and dogs with chronic kidney disease. J Vet Intern Med. (2010) 24:65–72. doi: 10.1111/j.1939-1676.2009.0426.x, PMID: 20041990

[45] Yan MT Chao CT Lin SH. Chronic kidney disease: strategies to retard progression. Int J Mol Sci. (2021) 22:10084. doi: 10.3390/ijms221810084, PMID: 34576247 PMC8470895

[46] Dunaevich A Chen H Musseri D Kuzi S Mazaki-Tovi M Aroch I. Acute on chronic kidney disease in dogs: Etiology, clinical and clinicopathologic findings, prognostic markers, and survival. J Vet Intern Med. (2020) 34:2507–15. doi: 10.1111/jvim.15931, PMID: 33044036 PMC7694831

[47] Bartges JW Willis AM Polzin DJ. Hypertension and renal disease. Vet Clin North Am Small Anim Pract. (1996) 26:1331–45. doi: 10.1016/s0195-5616(96)50131-2 8911022

[48] Parker VJ Freeman LM. Association between body condition and survival in dogs with acquired chronic kidney disease. J Vet Intern Med. (2011) 25:1306–11. doi: 10.1111/j.1939-1676.2011.00805.x, PMID: 22092621

