# Supplementary material for: Real-world data on canine chronic kidney disease in Greece: clinical and quality of life insights
Source: Front Vet Sci. 2025 Aug 11;12:1601044. doi: 10.3389/fvets.2025.1601044 (PMC12376200; doi:10.3389/fvets.2025.1601044)

**Appendix**

Table A1. Eligibility criteria for observational study

| Inclusion criteria | - The dog must have a veterinarian-confirmed diagnosis of CKD with any IRIS stage (i.e., Stage 1, 2, 3, or 4). - The dog must be stable and been treated for, or is currently being treated for, CKD. If stable and staged up to three months prior to recruitment, staging bloodwork is to be performed and the dog should stabilize before the owner completes the instruments. - The dog must be a minimum of three years of age. - The owner of the dog with CKD must be 18 years or above and the primary caretaker. - The owner of the dog with CKD is willing and able to answer written questions and instruments in Greek related to their dog with CKD at two different timepoints. |
| --- | --- |
| Exclusion criteria | - The dog is known or suspected to be pregnant or lactating. - The dog is eating an unbalanced diet. - The dog required medical hospitalization in the seven days immediately prior to the owner completed questionnaires. - The dog has any of the following medical conditions: - Heartworm disease - Uncontrolled hyperparathyroidism - Primary hyperparathyroidism or primary hypoparathyroidism - Protein-losing enteropathy - Polycystic kidney disease, renal dysplasia - Hypercalcemia of renal or non-renal secondary hyper parathyroid origin - Pyelonephritis currently, hospitalized with a diagnosis of pyelonephritis (however, if dogs with episodes longer than one year ago and have resolved, these dogs can participate) - Post-renal azotemia such as obstructive lower urinary tract disease/urolithiasis (canines with episodes longer than one year in the past are not excluded). - Any malignant or suspected malignant neoplasia. - Acute Kidney Injury (AKI) or acute azotemia. For example, do not enroll canines in uremic crisis (ethylene glycol toxicity, acute on chronic episodes, blocked cat) but once stable this canine can be enrolled in study at a future time when at a stable IRIS stage. - The canine is receiving treatment, or a history of requiring treatment in the last six months, for active infectious disease that secondarily causes nephropathology, such as Lyme nephritis, ehrlichiosis, leishmaniosis or leptospirosis. - The dog is enrolled in a clinical study in which an investigational veterinary product is used. |

Table A2. Description of the International Renal Interest Society (IRIS) staging guidelines in dogs

| Description of IRIS Stages | Stage 1: Blood Creatinine <125 µmol/l or <1.4 mg/dl, SDMA <18 µg/dl.  Normal blood creatinine or normal or mild increase blood SDMA. Some other renal abnormality present (such as, inadequate urinary concentrating ability without identifiable non-renal cause (in cats not dogs), abnormal renal palpation or renal imaging findings, proteinuria of renal origin, abnormal renal biopsy results, increasing blood creatinine or SDMA concentrations in samples collected serially). Persistently elevated blood SDMA concentration (>14 µg/dl) may be used to diagnose early CKD. |
| --- | --- |
|  | Stage 2: Blood Creatinine 125-250 µmol/l or 1.4-2.8 mg/dl, SDMA 18-35 µg/dl.  Normal or mildly increased creatinine, mild renal azotemia (lower end of the range lies within reference ranges for creatinine for many laboratories, but the insensitivity of creatinine concentration as a screening test means that patients with creatinine values close to the upper reference limit often have excretory failure). Mildly increased SDMA. Clinical signs usually mild or absent. |
|  | Stage 3: Blood Creatinine 250-440 µmol/l or 2.9-5.0 mg/dl, SDMA 36-54 µg/dl.  Moderate renal azotemia. Many extrarenal signs may be present, but their extent and severity may vary. If signs are absent, the case could be considered as early Stage 3, while presence of many or marked systemic signs might justify classification as late Stage 3. |
|  | Stage 4: Blood Creatinine >440 µmol/l or >5.0 mg/dl, SDMA >54 µg/dl.  Increasing risk of systemic clinical signs and uremic crises. |

Table A3. Veterinarian-completed form

| **Information** | **Question in full** | **Response options** |
| --- | --- | --- |
| Age | What is the dog’s age? | Free text |
| Sex | What is the dog’s sex? | - Male intact - Female intact - Male neutered - Female spayed |
| Breed | What is the dog's breed? | Free text |
| Weight | What is the dog’s weight? | Free text |
| Comorbidities | Indicate any diagnosed concomitant conditions for this dog | - Mitral valve disease - Congestive heart failure - Peripheral vascular disease - Cerebrovascular disease - Hemiplegia or paraplegia - Dementia - Degenerative Joint Disease/Arthritis - Stomach and intestinal ulcers - Diabetes - Any malignancy, including leukemia and lymphoma - Metastatic solid tumor - Mild liver disease - Moderate or severe liver disease - Hypertension - Anemia - Heart Murmur - Hypercalcemia - Periodontal disease - Cystitis - Urolithiasis - Cardiac abnormalities - Pathogenic infection - Obesity - Glomerulonephritis - Dermatitis - Atopy - No concomitant conditions - Other (specify) |

| Table A4. Case sheet of dog with chronic kidney disease: Animal Diet and Disease History**Information** | **Question in full** | **Response options** |
| --- | --- | --- |
| Dog info | Dog name | Free text |
| Dog info | Veterinarian Name | Free text |
| Animal diet | Record basic diet information (cooked foods, commercial, clinical diets, etc.) | Free text |
| Disease history | Record the animal’s basic medical history that may be related to CKD | Free text |

Table A5. Case sheet of dog with chronic kidney disease: Clinical signs and Most Troublesome Clinical signs

|  | **Clinical signs currently present** | **Most troublesome clinical signs** | |
| --- | --- | --- | --- |
|  |  | **For your dog** | **For you, when caring for your dog** |
| 1. Increased/excessive water consumption |  |  |  |
| 2.Increased/excessive urination |  |  |  |
| 3. Unexplained weight loss / loss of muscle mass / body condition |  |  |  |
| 4. Loss/decrease of appetite |  |  |  |
| 5. Dog is not eating at all |  |  |  |
| 6. Depressed mood |  |  |  |
| 7. Weakness/fatigue |  |  |  |
| 8. Vomiting |  |  |  |
| 9. Diarrhoea |  |  |  |
| 10. Halitosis / Very bad breath |  |  |  |
| 11. Pale gums |  |  |  |
| 12. Stomach / intestinal inflammation (gastroenteritis) |  |  |  |
| 13. Mouth ulcers |  |  |  |
| 14. Problems with vision |  |  |  |
| 15. Unkept appearance |  |  |  |
| 16. Fragile bones |  |  |  |
| 17. Blood in urine |  |  |  |
| 18. Other (please specify) __________________________ |  |  |  |
| 19. Other (please specify) __________________________ |  |  |  |
| 20. My dog has/had no clinical signs |  |  | |

Table A6. Owner-completed form

| **Demographics** | **Question in full** | **Response options** |
| --- | --- | --- |
| Gender | Are you: | Male; Female; Intersex |
| Age | What is your age? | Free text |
| Employment | What is your current main employment status? | - I work full time - I work part time - I’m a student - I’m not working because I choose not to - I’m unemployed - I’m not working because I am retired - I’m on long term sick leave |
| Employment | What is your highest level of education? | - Primary education (e.g. primary school - Secondary education (e.g. Gymnasium or Lyceum) - College or Associate degree (vocational or academic) - Undergraduate degree (Bachelor’s degree, Professional degree or equivalent) - Graduate degree (Masters, Doctorate or equivalent) - Not listed, please specify _______________________ |

Table A7. Categorization of reported concomitant diseases.

| **Cardiovascular** | Mitral valve disease, Congestive heart failure, Peripheral vascular disease, Hypertension, Heart Murmur, Cardiac abnormalities |
| --- | --- |
| **Musculoskeletal** | Degenerative Joint Disease/Arthritis, bilateral patellar dislocation |
| **Nervous system** | Cerebrovascular disease, Hemiplegia or paraplegia, Dementia, Polyneuritis, Idiopathic Epileptic Seizures |
| **Haematologic** | Anemia |
| **Renal disease/Urinary Tract** | Cystitis, Urolithiasis, Glomerulonephritis, Nephrolithiasis |
| **GI disease** | Stomach and intestinal ulcers |
| **Cancer** | Any malignancy, including leukemia and lymphoma, Metastatic solid tumor |
| **Dermatologic** | Dermatitis, Atopy |
| **Endocrine/metabolic** | Diabetes, Hypercalcaemia, Cushing’s Disease, Hypothyroidism |
| **Liver** | Mild liver disease, Moderate or severe liver disease, Mild Cholecystitis |
| **Periodontal disease** | Periodontal disease |
| **Pathogenic infection** | Pathogenic infection, Leishmaniosis |
| **Obesity** | Obesity |
| **Other** | Pigmentary Keratitis (one eye), Urinary incontinence, Surgical abscission of the left kidney (2 years ago), Breast Mass/surgical abscission, Amputated right hind leg, Prostate gland disease, Prostate gland hyperplasia, Mandibular lymph node oedema |

Table A8. Canine breeds by IRIS Stage

| **Breed** | **Stage1**  **N (%)** | **Stage 2**  **N (%)** | **Stage 3**  **N (%)** | **Stage 4**  **N (%)** | **Total**  **N (%)** |
| --- | --- | --- | --- | --- | --- |
| Mixed Breed | 18 (22.2) | 45 (55.6) | 11 (13.6) | 7 (8.6) | 81 37.7%) |
| Maltese | 4 (22.2) | 11 (61.1) | 1 (5.6) | 2 (11.1) | 18 (8.4%) |
| Yorkshire Terrier | 4 (26.7) | 9 (60.0) | 2 (13.3) | - (0.0) | 15 (7.0%) |
| Labrador Retriever | 2 (18.2) | 7 (63.6) | 2 (18.2) | - (0.0) | 11 (5.1%) |
| Jack Russell Terrier | 3 (37.5) | 3 (37.5) | 2 (25.0) | - (0.0) | 8 (3.7%) |
| Kokoni | 1 (14.3) | 3 (42.9) | 1 (14.3) | 2 (28.6) | 7 (3.3%) |
| Akita | 2 (40.0) | 3 (60.0) | - (0.0) | - (0.0) | 5 (2.3%) |
| Boxer | - (0.0) | 2 (40.0) | 2 (40.0) | 1 (20.0) | 5 (2.3%) |
| Pit Bull | - (0.0) | 3 (60.0) | 1 (20.0) | 1 (20.0) | 5 (2.3%) |
| American Cocker Spaniel | 1 (25.0) | 2 (50.0) | 1 (25.0) | - (0.0) | 4 (1.9%) |
| Bichon Frise | 1 (33.3) | 2 (66.7) | - (0.0) | - (0.0) | 3 (1.4%) |
| GSD | 2 (66.7) | 1 (33.3) | - (0.0) | - (0.0) | 3 (1.4%) |
| Miniature Pinscher | 1 (33.3) | 2 (66.7) | - (0.0) | - (0.0) | 3 (1.4%) |
| Poodle (Caniche) | - (0.0) | 3 (100.0) | - (0.0) | - (0.0) | 3 (1.4%) |
| Rottweiler | 1 (33.3) | 2 (66.7) | - (0.0) | - (0.0) | 3 (1.4%) |
| Shar Pei | 3 (100.0) | - (0.0) | - (0.0) | - (0.0) | 3 (1.4%) |
| West Highland White Terrier | 2 (66.7) | - (0.0) | - (0.0) | 1 (33.3) | 3 (1.4%) |
| English Setter | - (0.0) | 2 (100.0) | - (0.0) | - (0.0) | 2 (0.9%) |
| German Pinscher | 1 (50.0) | - (0.0) | 1 (50.0) | - (0.0) | 2 (0.9%) |
| Greek Griffon | - (0.0) | 2 (100.0) | - (0.0) | - (0.0) | 2 (0.9%) |
| Griffon belge | 1 (50.0) | 1 (50.0) | - (0.0) | - (0.0) | 2 (0.9%) |
| Rhodesian Ridgeback | 2 (100.0) | - (0.0) | - (0.0) | - (0.0) | 2 (0.9%) |
| Belgian Shepherd Dog | - (0.0) | 1 (100.0) | - (0.0) | - (0.0) | 1 (0.5%) |
| Bobtail | - (0.0) | 1 (100.0) | - (0.0) | - (0.0) | 1 (0.5%) |
| Border Collie | - (0.0) | - (0.0) | 1 (100.0) | - (0.0) | 1 (0.5%) |
| Bull Terrier | - (0.0) | - (0.0) | - (0.0) | 1 (100.0) | 1 (0.5%) |
| Cane Corso | - (0.0) | - (0.0) | 1 (100.0) | - (0.0) | 1 (0.5%) |
| Caniche-Terrier | - (0.0) | 1 (100.0) | - (0.0) | - (0.0) | 1 (0.5%) |
| Caucasian Shepherd Dog | - (0.0) | - (0.0) | 1 (100.0) | - (0.0) | 1 (0.5%) |
| Cavalier King Charles Spaniel | - (0.0) | 1 (100.0) | - (0.0) | - (0.0) | 1 (0.5%) |
| Chow Chow | 1 (100.0) | - (0.0) | - (0.0) | - (0.0) | 1 (0.5%) |
| Dalmatian | - (0.0) | - (0.0) | 1 (100.0) | - (0.0) | 1 (0.5%) |
| Doberman | - (0.0) | 1 (100.0) | - (0.0) | - (0.0) | 1 (0.5%) |
| Dogo Argentino | - (0.0) | 1 (100.0) | - (0.0) | - (0.0) | 1 (0.5%) |
| English Cocker Spaniel | - (0.0) | 1 (100.0) | - (0.0) | - (0.0) | 1 (0.5%) |
| English Pointer | - (0.0) | 1 (100.0) | - (0.0) | - (0.0) | 1 (0.5%) |
| English Springer Spaniel | - (0.0) | 1 (100.0) | - (0.0) | - (0.0) | 1 (0.5%) |
| Epaniel Breton | - (0.0) | 1 (100.0) | - (0.0) | - (0.0) | 1 (0.5%) |
| French Bulldog | - (0.0) | 1 (100.0) | - (0.0) | - (0.0) | 1 (0.5%) |
| German Spitz-Miniature | - (0.0) | 1 (100.0) | - (0.0) | - (0.0) | 1 (0.5%) |
| Miniature Bull Terrier | 1 (100.0) | - (0.0) | - (0.0) | - (0.0) | 1 (0.5%) |
| Pekingese | 1 (100.0) | - (0.0) | - (0.0) | - (0.0) | 1 (0.5%) |
| Pomeranian | - (0.0) | 1 (100.0) | - (0.0) | - (0.0) | 1 (0.5%) |
| Pug | - (0.0) | - (0.0) | 1 (100.0) | - (0.0) | 1 (0.5%) |
| Shih Tzu | - (0.0) | 1 (100.0) | - (0.0) | - (0.0) | 1 (0.5%) |
| Siberian Husky | 1 (100.0) | - (0.0) | - (0.0) | - (0.0) | 1 (0.5%) |
| Toy Poodle | - (0.0) | 1 (100.0) | - (0.0) | - (0.0) | 1 (0.5%) |
| Grand Total | 53 | 118 | 29 | 15 | 215 (100.0%) |

Table A9. Estimated number of dogs below the geriatric age with cCKD reported per Weight Group.

| **Weight Groups** | **<6.75 Kgs** | **6.75 - <11.25 Kgs** | **11.25 - <20.25 Kgs** | **20.25 - <29.25 Kgs** | **>29.25 Kgs** |
| --- | --- | --- | --- | --- | --- |
| **Mean age** | 12.9 | 12.0 | 9.4 | 9 | 7.9 |
| **Geriatric age (UC Davis)** | 11 | 11 | 9 | 8 | 7 |
| **Approximate number of cases below geriatric age** | 11/57 | 14/54 | 14/33 | 11/35 | 11/36 |

Table A10. List of Comorbidities reported

| **Comorbidities** | **Total Ν** |
| --- | --- |
| No comorbidities | 57 |
| Periodontal disease | 57 |
| Degenerative joint disease/Arthritis | 39 |
| Mitral valve disease | 35 |
| Heart murmur | 28 |
| Mild liver disease | 24 |
| Anemia | 20 |
| Dermatitis | 13 |
| Moderate or severe liver disease | 12 |
| Congestive heart failure | 12 |
| Hypertension | 11 |
| Obesity | 11 |
| Atopy | 10 |
| Urolithiasis | 9 |
| Cystitis | 9 |
| Glomerulonephritis | 8 |
| Cardiac abnormalities | 8 |
| Stomach and intestinal ulcers | 6 |
| Any malignancy, including leukemia and lymphoma | 6 |
| Dementia | 5 |
| Canine Leishmaniosis* | 5 |
| Pathogenic infection | 4 |
| Cushing’s Disease | 4 |
| Hypothyroidism | 3 |
| Hypercalcaemia | 2 |
| Metastatic solid tumor | 2 |
| Mild cholecystitis | 1 |
| Nephrolithiasis | 1 |
| Polyneuritis | 1 |
| Hemiplegia or paraplegia | 1 |
| Idiopathic Epileptic Seizures | 1 |
| Bilateral patellar dislocation | 1 |
| Diabetes | 1 |
| Pigmentary Keratitis (one eye) | 1 |
| Urinary incontinence | 1 |
| Surgical abscission of the left kidney, 2 years ago | 1 |
| Breast Mass/surgical abscission | 1 |
| Amputated right hind leg | 1 |
| Prostate gland disease | 1 |
| Prostate gland hyperplasia | 1 |
| Mandibular lymph node oedema | 1 |

*reported as a comorbidity by the practitioners aligned with the inclusion/exclusion criteria (e.g. received treatment for Canine Leishmaniosis more than 6 months ago)

Table A11. Clinical signs of dogs with cCKD, as reported by the practitioners.

| **Clinical signs reported** | **Stage 1**  **N (%)** | **Stage 2**  **N (%)** | **Stage 3**  **N (%)** | **Stage 4**  **N (%)** | **Total**  **N (%)** |
| --- | --- | --- | --- | --- | --- |
| Increased/excessive urination | 21 (39.6) | 59 (50.0) | 19 (65.5) | 11 (73.3) | 110 (51.2) |
| Halitosis / Very bad breath | 22 (41.5) | 62 (52.5) | 13 (44.8) | 13 (86.7) | 110 (51.2) |
| Increased/excessive water consumption | 18 (34.0) | 60 (50.8) | 18 (62.1) | 11 (73.3) | 107 (49.8) |
| Weakness/fatigue | 21 (39.6) | 44 (37.3) | 14 (38.3) | 9 (60.0) | 88 (40.9) |
| Loss/decrease of appetite | 12 (22.6) | 45 (38.1) | 10 (34.5) | 8 (53.3) | 75 (34.9) |
| Unexplained weight loss / loss of muscle mass / body condition | 9 (17.0) | 34 (28.8) | 11 (37.9) | 6 (40.0) | 60 (27.9) |
| Vomiting | 9 (17.0) | 31 (26.3) | 10 (34.5) | 7 (46.7) | 57 (26.5) |
| Problems with vision | 9 (17.0) | 20 (16.9) | 4 (13.8) | 5 (33.3) | 38 (17.7) |
| Depressed mood | 10 (18.9) | 13 (11.0) | 2 (6.9) | 8 (53.3) | 33 (15.4) |
| Pale gums | 2 (3.8) | 13 (11.0) | 5 (17.2) | 5 (33.3) | 24 (11.2) |
| Diarrhoea | 3 (5.7) | 12 (10.2) | 5 (17.2) | 3 (20.0) | 23 (10.7) |
| Stomach/intestinal inflammation (gastroenteritis) | 2 (3.8) | 10 (8.5) | 1 (3.4) | 4 (26.7) | 17 (7.9) |
| Unkept appearance | 4 (7.5) | 8 (6.8) | 1 (3.4) | 3 (20.0) | 16 (7.4) |
| Dog is not eating at all | 1 (1.9) | 7 (5.9) | 2 (6.9) | 3 (20) | 13 (6.1) |
| Blood in urine | 1 (1.9) | 10 (8.5) | 2 (6.9) | 0 (0.0) | 13 (6.1) |
| Fragile bones | 1 (1.9) | 2 (1.7) | 2 (6.9) | 0 (0.0) | 5 (2.3) |
| Mouth ulcers | 1 (1.9) | 2 (1.7) | 0 (0.0) | 1 (6.7) | 4 (1.9) |
| Total | 53 (24.7) | 118 (54.9) | 29 (13.5) | 15 (7.0) | 215 (100.0) |

Table A12. Point-biserial correlation between presence of clinical signs and severity of cCKD according to IRIS Stage. The statistically significant results are depicted with an asterisk.

| **Clinical signs** | **correlation coefficient** | **p-value** |
| --- | --- | --- |
| Increased/excessive water consumption | 0,22 | *0,001 |
| Increased/excessive urination | 0,19 | *0,004 |
| Unexplained weight loss / loss of muscle mass / body condition | 0,16 | *0,021 |
| Loss/decrease of appetite | 0,14 | *0,036 |
| Dog is not eating at all | 0,16 | *0,019 |
| Depressed mood | 0,11 | 0,100 |
| Weakness/fatigue | 0,10 | 0,146 |
| Vomiting | 0,17 | *0,011 |
| Diarrhea | 0,14 | *0,046 |
| Halitosis / Very bad breath | 0,16 | *0,019 |
| Pale gums | 0,22 | *0,001 |
| Stomach / intestinal inflammation (gastroenteritis) | 0,14 | *0,043 |
| Mouth ulcers | 0,04 | 0,583 |
| Problems with vision | 0,06 | 0,388 |
| Unkept appearance | 0,06 | 0,416 |
| Fragile bones | 0,03 | 0,634 |
| Blood in urine | 0,02 | 0,823 |

Table A13. Clinical signs reported by the owner as more troublesome for the owner and the dog. The statistically significant results are depicted with an asterisk.

| **Clinical signs** | **Troublesome for the dog (%, 95%CI)** | **Troublesome for the owner (%, 95%CI)** | **Total** | **Fischer’s exact test p-value** |
| --- | --- | --- | --- | --- |
| Increased/excessive urination | 34 (30.9, 23.0-40.1) | 48 (43.6, 34.7-53.0) | 110 | 0.0696 |
| Halitosis / Very bad breath | 18 (16.4, 10.6-24.4) | 60 (54.5, 45.2-63.5) | 110 | *<0.001 |
| Increased/excessive water consumption | 32 (29.9, 22.1-39.2) | 27 (25.2, 18.0-34.2) | 107 | 0.5408 |
| Weakness/fatigue | 39 (44.3, 34.4-54.7) | 17 (19.3, 12.4-28.8) | 88 | *<0.001 |
| Loss/decrease of appetite | 30 (40.0, 29.7-51.3) | 31 (41.3, 30.9-52.6) | 75 | 1.0 |
| Unexplained weight loss / loss of muscle mass / body condition | 20 (33.3, 22.7-45.9) | 15 (25.0, 15.8-37.2) | 60 | 0.4220 |
| Vomiting | 34 (59.6, 46.7-71.4) | 30 (52.6, 39.9-65.0) | 57 | 0.5715 |
| Problems with vision | 20 (52.6, 37.3-67.5) | 5 (13.2, 5.8-27.3) | 38 | *<0.001 |
| Depressed mood | 11 (33.3, 19.8-50.4) | 9 (27.3, 15.1-44.2) | 33 | 0.7893 |
| Pale gums | 6 (25.0, 12.0-44.9) | 6 (25.0, 12.0-44.9) | 24 | 1.0 |
| Diarrhea | 18 (78.3, 58.1-90.3) | 12 (52.2. 33.0-70.8) | 23 | 0.1205 |
| Stomach/intestinal inflammation (gastroenteritis) | 8 (47.1, 26.2-69.0) | 7 (41.2, 21.6-64.0) | 17 | 1.0 |
| Unkept appearance | 2 (12.5, 3.5-36.0) | 7 (43.8, 23.1-66.8) | 16 | 0.1134 |
| Dog is not eating at all | 6 (46.2, 23.2-70.9) | 4 (30.8, 12.7-57.6) | 13 | 0.6882 |
| Blood in urine | 6 (46.2, 23.2-70.9) | 5 (38.5, 17.7-64.5) | 13 | 1.0 |
| Fragile bones | 4 (80.0, 37.6-96.4) | 0 (0.0, 0.0-43.4) | 5 | *0.0476 |
| Mouth ulcers | 3 (75.0, 30.1-95.4) | 0 (0.0, 0.0-49.0) | 4 | 0.1429 |

Table A14. Descriptive statistics of the biochemical examinations performed in the study population

| **Parameter** | **Mean** | **Standard Deviation** | **Minimum** | **Maximum** | **Reference Range** |
| --- | --- | --- | --- | --- | --- |
| bCREA | 2.275 | 1.491 | 0.58 | 8.97 | 0.50 - 1.40 |
| ALB | 2.775 | 0.478 | 1.40 | 4.40 | 3.2 - 4.7 |
| TP | 7.251 | 1.109 | 5.40 | 12.50 | 5.3 - 7.6 |
| P | 6.219 | 3.574 | 1.90 | 22.20 | 2.6 - 6.2 |
| Ca | 10.185 | 1.111 | 7.70 | 13.70 | 8.5 - 11.9 |
| Ca++ | 1.294 | 0.135 | 1.00 | 1.87 | 1.12 - 1.50 |
| K | 5.182 | 0.756 | 3.10 | 7.50 | 3.70 - 5.80 |
| Na | 146.360 | 5.160 | 129.00 | 173.00 | 140 - 155 |
| Cl | 107.782 | 5.587 | 76.00 | 137.00 | 98.0 - 107.0 |
| BUN | 59.103 | 46.760 | 9.00 | 327.00 | 10.0 - 32.0 |
| Na/K ratio | 28.821 | 4.142 | 18.75 | 45.16 |  |
| Up/C ratio | 1.27 | 1.73 | 0.01 | 12.47 |  |

Table A15. Owners’ demographics

| **Characteristic** | **Category** | **Male** | **Female** | **Total** |
| --- | --- | --- | --- | --- |
| **Total Respondents** |  | 75 | 140 | 215 |
| **Age Statistics** |  |  |  |  |
|  | Mean Age (years) | 45.9 | 47.5 |  |
|  | Median Age (years) | 45.0 | 47.5 |  |
|  | Standard Deviation | 12.9 | 12.7 |  |
|  | Age Range (years) | 22 - 76 | 20 - 77 |  |
| **Employment Status** |  | N (%) | N (%) | N (%) |
|  | I work full time | 56 (74.7%) | 80 (57.1%) | 136 (63.3%) |
|  | I work part time | 2 (2.7%) | 16 (11.4%) | 18 (8.4%) |
|  | I’m a student | 2 (2.7%) | 2 (1.4%) | 4 (1.9%) |
|  | I’m not working because I am retired | 10 (13.3%) | 22 (15.7%) | 32 (14.9%) |
|  | I’m unemployed | 5 (6.0%) | 13 (9.3%) | 18 (8.4%) |
|  | I’m not working because I choose not to | 0 (0.0%) | 7 (5.0%) | 7 (3.3%) |
| **Education Level** |  | N (%) | N (%) | N (%) |
|  | Primary education | 0 (0.0%) | 3 (2.1%) | 3 (1.4%) |
|  | Secondary education | 23 (30.7%) | 53 (37.9%) | 76 (35.3%) |
|  | College or Associate degree | 19 (25.3%) | 23 (16.4%) | 42 (19.5%) |
|  | Undergraduate degree | 19 (25.3%) | 38 (27.1%) | 57 (26.5%) |
|  | Graduate degree | 13 (17.3%) | 23 (16.4%) | 36 (16.8%) |
|  | N/A | 1 (1.3%) | 0 (0.0%) | 1 (0.5%) |

Figure A1. Histogram and kernel density estimation (KDE) for Canine Age, showing the Free Probability Density Function (PDF) for the normal distribution

.
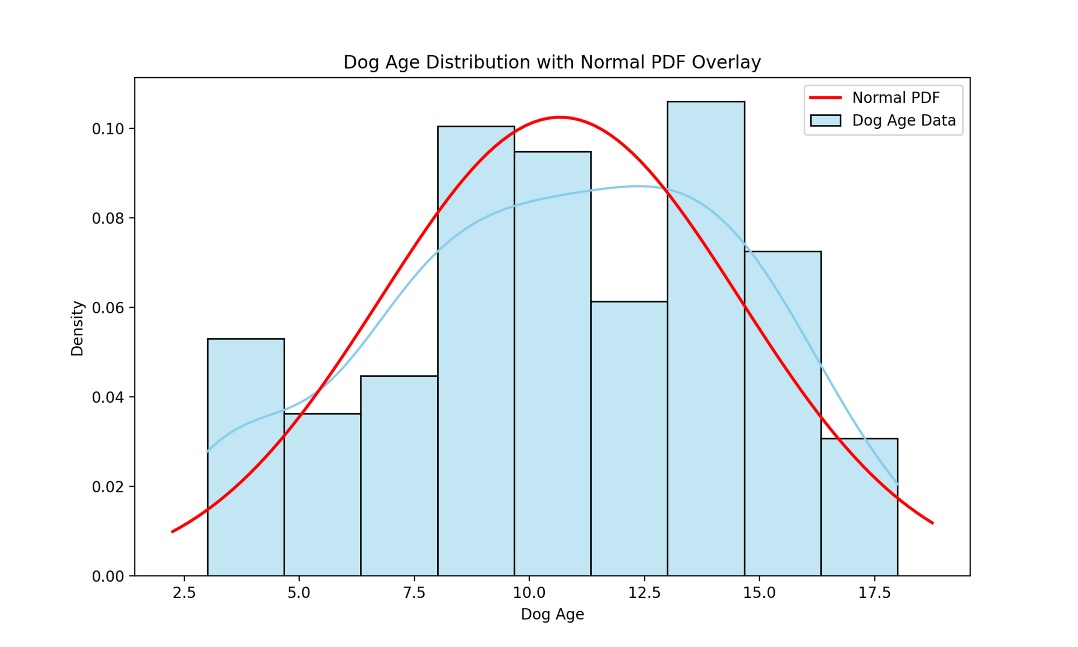

Figure A2. Shapiro-Wilk p-values by IRIS Stage and Measure


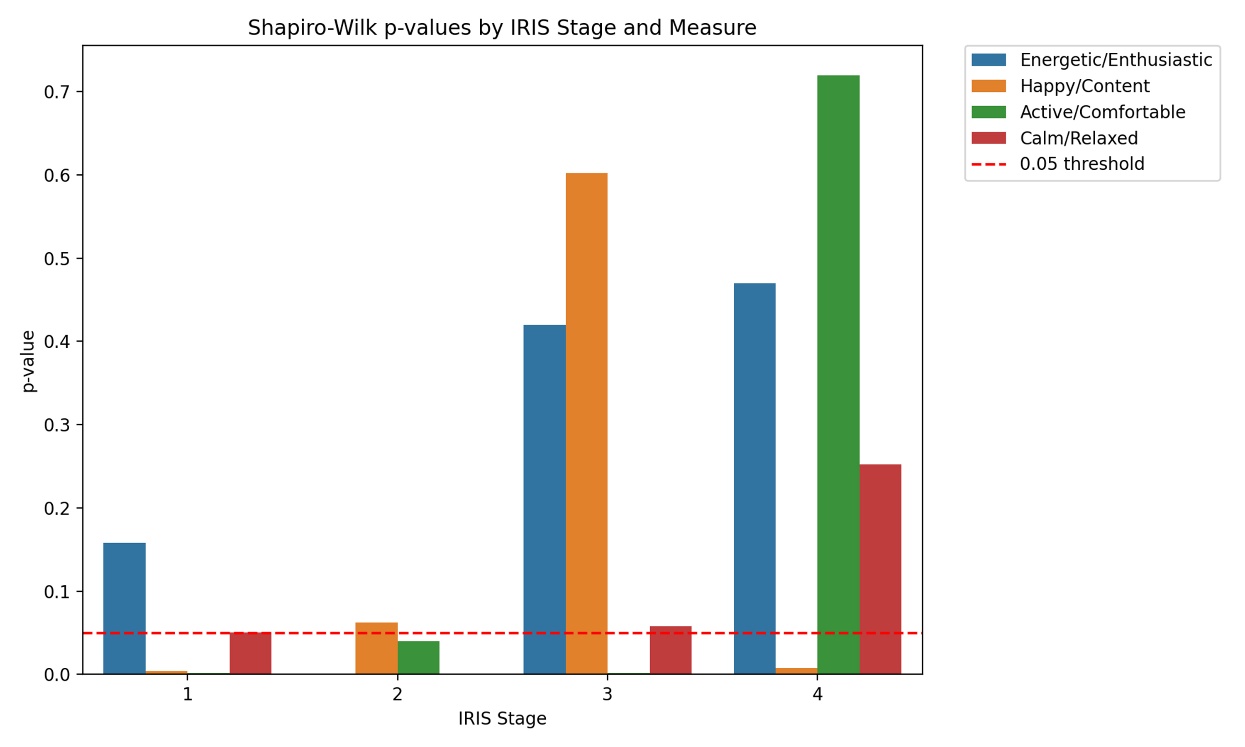

Supplement: Supplementary file 1 [file Data_Sheet_1.docx]
